# Epigenetic mechanisms behind cellular sensitivity to DNA damage

**DOI:** 10.15698/cst2018.07.145

**Published:** 2018-06-12

**Authors:** Amanda K. Williamson, Zijing Zhu, Zhi-Min Yuan

**Affiliations:** 1 Department of Environmental Health, John B. Little Center for Radiation Sciences, Harvard T.H. Chan School of Public Health, Boston, MA, USA.

## Abstract

Epigenetic regulation of gene expression in cells is a complex and dynamic process that remains incompletely understood. The architecture of the chromatin itself and its level of condensation can greatly impact the expression of genes as well as the sensitivity of the DNA to damage. The compact nature of heterochromatin typically results in gene silencing and resistance to DNA-damaging agents, while less compact euchromatin results in gene expression and increased sensitivity to injury. There are diverse ways in which the chromatin structure, and therefore the sensitivity of cells to damage, can be regulated, including post-translational modifications to both the histones within the chromatin and the DNA itself. These modifications are tightly controlled and correspond to various factors such as metabolism and cell cycle. When these processes are dysregulated, as in cancer cells, the chromatin structure is also altered, ultimately changing the gene expression profile as well as the susceptibility of cells to DNA-damaging agents commonly used for cancer treatments. Recent studies have shown that manipulating the various players involved in regulating post-translational modifications to chromatin and exploiting differences in metabolism may prove to be effective methods for modifying cancer and normal cell sensitivity to damaging agents. In this review we discuss various ways of regulating chromatin structure and how these changes can influence cellular sensitivity to damage as well as the implications of these relationships for improving the efficacy and safety of cancer treatments.

## INTRODUCTION

Epigenetics is the regulation of gene expression independent of the gene sequence itself via the interactions between DNA and proteins called histones 1. In the nucleus, DNA is highly compacted by wrapping around four major histones, H2A, H2B, H3 and H4, to form chromatin. These histones, bound to DNA, arrange into an octamer to create a nucleosome 1,2. The level of chromatin compaction within these structures determines the accessibility of genes to cellular machinery necessary for their transcription into mRNA that ultimately gets translated into proteins. The higher order chromatin structure also impacts the degree of exposure of the DNA to damaging agents and repair mechanisms 2,3. Thus, modulating the interactions between DNA and histones via post-translational modifications represents a method of regulating gene expression 1,3.

There are two major types of chromatin: heterochromatin, a transcriptionally inactive form in which DNA is compacted, and euchromatin, a transcriptionally active form in which DNA is more relaxed 3. Chromatin compaction is regulated by post-translational modifications to histones and DNA, including methylation, acetylation, phosphorylation and ubiquitination. Methylation of DNA and histone tails is a hallmark of chromatin compaction, as heterochromatin is distinguished by tri-methylation on lysine 27 of histone H3 (H3K27me) and di-/trimethylation on lysine 9 of histone H3 (H3K9me). However, methylation patterns are context-dependent, as trimethylation of lysine 4 on H3 (H3K4me3) results in gene activation. Conversely, acetylation of basic lysine and arginine residues on histones reduces their positive charge, and therefore decreases their binding affinity to the negatively charged backbone of DNA, resulting in a more relaxed chromatin state 4. Euchromatin is characterized by hyperacetylation of histones, including acetylation of histone H3 lysine 9 and H4 N-terminal lysines 3,5.

These post-translational modifications are heritable and used to control gene expression, as well as to protect DNA from mutation. The higher order chromatin structure is extremely important for maintaining genome stability at key points in the genome 5. For example, DNA is more highly methylated, and therefore compacted, in areas that contain repetitive sequences to prevent the expression of non-coding DNA, induction of double-stranded breaks (DSBs), transposable elements and inappropriate recombination events likely to occur at repetitive DNA sequences. These events can lead to mutations, genome instability and cancer development 5,6. In this way, various combinations of modifications to histones and DNA alter chromatin structure in order to regulate the global gene expression pattern in a cell and can vary based on cell type. Often times, this precise chromatin architecture at specific gene sequences, including the promoter regions of oncogenes and tumor suppressors, is altered in cancer cells 6. Chromatin compaction and relaxation is a dynamic and integrated process dependent upon many factors, including cell status and metabolism. In this context, regulation of DNA architecture may prove to be a novel method to alter cells’ susceptibility to DNA-damaging agents used in cancer therapies.

## CHROMATIN STATUS IMPACTS CELLULAR SENSITIVITY TO DNA DAMAGE 

Chromatin that is more compact, as in heterochromatin, is more resistant to DNA damage and protected from DSBs, while chromatin that is less compact is more sensitive to damage 5,7. A recent study found that decondensed chromatin exhibited a 5-50-fold increase in radiation-induced double-stranded DNA breaks 7. Heterochromatin is refractory to DSBs because its compact nature limits DNA exposure to damaging agents and surrounding non-histone proteins in the nucleosome structure provide physical protection to the DNA 3,5. However, chromatin plasticity is critical in order to repair DNA breaks induced by external stressors and varies based on the chromatin architecture. When DNA damage occurs, the nucleosome structure becomes disrupted and chromatin is decompacted through various types of histone modifications, including destabilization of the nucleosome by incorporation of the H2A.Z histone to replace H2A, as well as ubiquitination and subsequent removal of H3 and H4. Additionally, there is often increased H4K5 acetylation and decreased H3K9 dimethylation to further open the chromatin structure at the site of the lesion. This extensive decompaction process and H4 hyperacetylation triggers the accumulation and docking of repair proteins at the site of the break, such as Ataxia Telangiectasia Mutation (ATM). ATM plays a role in identifying the specific lesion and triggering its repair through various mechanisms including non-homologous end joining (NHEJ) and homologous recombination (HR) 5,8. However, heterochromatin is often seen as being refractory to DNA repair if damage does occur because of its condensed nature preventing access to repair proteins. It has also been shown to be more reliant upon ATM-induced chromatin relaxation in order to effectively reduce the heterochromatic structure to allow for repair 8, thus the specific type of chromatin remodeling that occurs in response to DNA damage remains context-dependent 5. Overall, epigenetic modifications to the chromatin architecture play a major role in regulating the susceptibility of DNA to damage as well as the ability of the cell to repair that damage.

## THE INFLUENCE OF CELL METABOLISM ON CHROMATIN STRUCTURE

One way that chromatin state and cellular sensitivity to damage can be modulated is through the direct interplay between the chromatin architecture and the metabolic state of a cell. Various enzymes play a role in the regulation of chromatin compaction, including DNA methyltransferases, such as EZH2, and histone deacetylases (HDACs). Methyltransferases are protein complexes with enzymatic activity that catalyze the addition of methyl groups onto arginine and lysine residues on histones. This methylation increases the binding affinity between the negatively charged backbone of DNA and the positive charge on the histones, resulting in chromatin compaction and reduction of gene transcription. Additionally, HDACs remove acetyl groups, which are responsible for maintaining a more open structure, further allowing the chromatin to become more compact 5.

Many of the enzymes that alter chromatin structure are dependent upon cellular metabolites and nutrient accessibility for their substrates and co-factors in order to catalyze enzymatic modifications to histones and DNA. This is one major way that chromatin dynamics respond to the environment and alter gene expression 5,9. For example, histone methyltransferases catalyze the transfer of a methyl group from S-adenosyl methionine, which is derived from the essential amino acid methionine and must be obtained via the diet through folate, vitamin B and choline, because they all carry a methyl group 4,9. Deficiencies in vitamin B12 can result in DNA hypomethylation in the promoter regions of some genes and has been linked to an increased risk of various cancers. A deficiency of methyl donor molecules has also been shown to impact DNA methylation patterns and gene expression in fetal development, demonstrating the impact of nutrient availability and metabolism on epigenetic regulation of gene expression 4.

Conversely, acetyl-CoA is a substrate of histone acetyltransferases (HATs) and is highly produced during aerobic glycolysis, generating increasingly acetylated histones and less compact chromatin under this metabolic state 9. Cancer cells are known to perform an increased rate of aerobic glycolysis, generating high levels of acetyl-CoA, as well as triggering the activation of AKT and MYC. This phenomenon is known as the Warburg effect. The rise of these metabolites and pathways increases the activity of HATs to acetylate histones, opening the DNA and promoting the transcription of growth-related genes. This process links changes in cellular metabolism to altered chromatin architecture that ultimately results in the modified gene expression profiles in cancer cells 1,9,10.

Additionally, histone demethylases including JMDH2 and TET2 require α-ketoglutarate generated from the TCA cycle as a substrate, as well as oxygen and Fe (II), in order to oxidize methylcytosines on histones to hydroxymethylcytosines and promote the removal of a methyl group 1,9. This mechanism is implicated during cell differentiation in order to activate differentiation-associated genes. Mutations in this pathway can cause hypomethylation of these genes, preventing their transcription and resulting in a hyperproliferative phenotype characteristic of tumors 9. α-ketoglutarate is derived from both glucose and glutamine taken in by the cell for generation of ATP, thus further demonstrating the influence of the cellular metabolic profile and the availability of metabolites from the environment on the ability of enzymes to alter the chromatin status and regulate epigenetics 1,9.

Cell type and cell state, such as pluripotent versus differentiated or primary versus metastatic cancer cells, are also directly related to chromatin design. The chromatin modification profile of a cell determines its phenotypic properties and the transition between cell states is dependent upon changes to the epigenomic profile 6,11. Embryonic stem cells contain both activating and repressive histone modifications at the site of promoters of genes specifically related to development. These modifications can be altered in stem cells at different developmental stages to either induce or silence gene expression, whereas differentiated cells have large areas of repressive H3Kme2 to maintain the expression of genes in accordance with a specific cell type 6. Cellular metabolism greatly influences the accessibility of components needed by enzymes to alter chromatin and thus determines whether a cell can transition between these different states. For example, reduced levels of NAD^+ ^due to glycolysis decreases the activity of NAD^+^-dependent histone deacetylases, resulting in increased gene expression and a cancerous phenotype. Its availability is also associated with chromatin compaction and gene silencing during the differentiation of mouse muscle stem cells 11.

Another determining factor of chromatin state is the cell cycle. Dividing cells rapidly replicate their DNA and chromatin configurations through a complex process of chromatin remodeling. When a cell is in S-phase of the cell cycle, its DNA must decondense and the nucleosome structures are reduced in order to open the DNA and allow the replication machinery to access the genes through tightly regulated processes 5,12; however, histones must also be replicated and properly reoriented with accurate post-translational modifications. If this order is not maintained, DNA can later undergo inappropriate transcription, leading to the formation of DNA-RNA hybrids, R-loops and increasing the likelihood of DNA to be exposed to breaks 5. Thus, rapidly dividing cells are much more susceptible to DNA breaks and the formation of improper chromatin structures, leading to the activation and/or silencing of incorrect genes. In contrast, a non-dividing cell that is in the gap phases of the cell cycle typically has more condensed DNA and stable nucleosome presence 12. As previously discussed, this condensation protects the DNA from DSBs 7. Due to the compact nature of heterochromatin in non-dividing cells and tissues consisting of non-dividing cells, they are more resistant to DNA damage. Variations in the metabolic programs and status of different cell types determine the level of chromatin compaction and exposure of DNA to replication errors, which determines the cells’ genomic integrity and sensitivity to DNA damage. It is this dynamic regulatory loop based upon cell cycle and metabolism that is implicated in various epigenetic changes in cancer cells 6.

## CANCER CELL METABOLISM INFLUENCES DNA ARCHITECTURE

Epigenetics has been shown to play a major role in the initiation and propagation of cancer cells due to its influence on the expression of oncogenic and tumor suppressor genes 6. Cancer cells are characterized by rapid cell division and deregulated metabolic programs centered around increasing the production of metabolites necessary for rapid growth and division. This unique cancer cell metabolic profile impacts chromatin structure within the cell 10. Because of their rapidly-dividing nature, cancer cells typically have less compact DNA, leading to overall genomic instability and increased likelihood of chromosome rearrangements. Additionally, their chromatin is characterized by global hypomethylation and acetylation of histones in promoter regions of genes that regulate growth and transformation, resulting in upregulation of growth-promoting and glycolytic genes 6,10. Cancer cells will also often exhibit epigenetic silencing of tumor suppressor proteins, DNA repair genes and transcription factors due to hypermethylation at the site of their promoters. These epigenetic alterations to gene expression can result in rapid cell growth and therefore accumulation of many mutations observed in cancer cells 6.

Epigenetic regulators are also commonly dysregulated in cancers. For example, EZH2 is frequently overexpressed in breast and prostate cancers, causing increased H3K27 methylation, chromatin compaction and transcriptional repression of tumor suppressor genes. This variation in cancer cells is one way in which deregulated epigenetic changes may contribute towards their malignant phenotype 6.

Additionally, studies have shown that epigenetic alterations observed in various cancers may be due to the initiation of these changes in normal and progenitor cancer stem cells. Methylation of genes involved in regulating self-renewal "gatekeeper" genes can allow progenitor cells to develop infinite self-renewal capacity and therefore be selected for survival and further replication, leading to accumulation of mutations and tumorigenesis. This epigenetic profile generates transformed stem-cell-like cancer cells with a high rate of proliferation 6.

The frequently observed decondensation of chromatin in rapidly-dividing cancer cells may be a beneficial aspect to target for therapy. The less compact chromatin structure allows for increased exposure, and therefore susceptibility of the chromatin to DNA-damaging agents commonly used in cancer therapies. Because epigenetics can be modified and potentially reversed in order to manipulate gene expression, it may prove to be advantageous to target epigenetic regulators in order to both alter the expression of tumor-suppressor and oncogenic genes in cancer cells as well as influence their vulnerability to DNA-damaging treatments 6. Chromatin dynamics in general are often dysregulated in various different ways in cancers that, when combined with additional metabolic alterations and genetic mutations, contribute to modifications in gene expression. Thus, epigenetics is one major factor in a complex and often context-dependent network of elements that lead to a cancerous phenotype in cells.

## CONCLUSION: IMPLICATIONS OF EPIGENETIC REGULATION FOR IMPROVED CANCER TREATMENT

Exploiting chromatin architecture regulation in order to manipulate cancer cell response to treatments is a therapeutic potential that requires further research. Due to the unique metabolic state of cancer cells, they rely on specific epigenetic profiles that could be novel targets for treatments. Because compact chromatin in heterochromatin is less susceptible to DNA damage and more favorable for survival 5, regulation of the chromatin architecture via manipulation of enzymes involved in modifying histones may be a useful strategy to increase cancer cell sensitivity to DNA-damaging agents. DSBs in heterochromatin are less common because the DNA is compact and protected, but they are also less likely to be repaired or undergo slow repair and therefore prove to be more deleterious 3,5. Additionally, DSBs initiate decondensation of chromatin in order to allow for repair 5,7, which would increase the accessibility of chromatin to further damage. Therefore, either reducing the compaction of chromatin to expose more DNA to damage or targeting the damage effect to heterochromatin in cancer cells could be a powerful way to increase cell death.

Recently, our lab has demonstrated that the level of the DNA methyltransferase, EZH2, is a determinant of tissue sensitivity to chemotherapy and radiation due to the level of p53 in renewable tissues 13. EZH2 is the catalytic component of Polycomb Repressor Complex 2 (PRC2), which trimethylates histone 3 at lysine 27, condensing chromatin and repressing many genes 14. It has been shown that sensitive tissues with high rates of replication often have high levels of p53 15, which contradicts the notion that p53 downregulation is necessary for proliferation 16,17. Thus, our lab sought to identify an indirect p53-mediated molecular mechanism behind the apparent sensitivity in renewable tissues to chemotherapeutic agents.

Because these tissues expressed high levels of p53, they also had high levels of its transcriptional target, MDM2. It had been previously determined that MDM2 physically associates with EZH2 14; therefore, our lab explored this interaction further by demonstrating that MDM2, when in a complex with MDMX, acts as a novel E3-ligase for EZH2 and leads to its degradation. Because sensitive tissues such as the spleen and thymus displayed an increased turnover of EZH2, their chromatin was less compact and more susceptible to damage from chemotherapy and radiation. Conversely, when p53 was mutated and transcriptionally inactive in these tissues, MDM2 levels were diminished, while EZH2 levels were increased in concomitance with increased H3K27me3, chromatin compaction, and protection against radio- and chemotherapy. Our novel findings for the role of EZH2 in tissue sensitivity demonstrate a previously unrecognized mechanism and possible target for protecting and/or sensitizing tissues to cancer therapies by manipulating epigenetic modifiers 13.

By targeting enzymes involved in chromatin structure modification, the status of DNA in cancer cells and normal cells can be altered to regulate tissue sensitivity to treatments. Targeting methyltransferases or deacetylases to reduce their activity in cancer cells would make the DNA less compact and increase its susceptibility to damage. Additionally, maintaining chromatin compaction in normal cells could protect them from DNA damage and unfavorable toxicity from chemotherapeutics. Combinational therapies that utilize inhibitors of overactive methyltransferases in cancer cells to open chromatin in conjunction with DNA-damaging agents such as gamma-rays, heavy ions and chemicals could enhance cancer cell-killing. Conversely, stabilizing methyltransferases in normal cells to maintain their chromatin compaction and genome integrity could protect these cells from adverse side effects of using DNA-damaging agents to kill cancer cells. Overall, modulation of epigenetic enzymes to alter chromatin structure in normal and cancer cells represents a previously understudied avenue of therapeutic potential for cancer treatments that may prove to be extremely beneficial in the future.

## Abbreviations

ATM: Ataxia Telangiectasia Mutation
DSB: double-stranded break
HDAC: histone deacetylase
HAT: histone acetyltransferase
